# Identifying a novel region in the Tembusu virus NS5 protein antagonizing type I interferon signaling

**DOI:** 10.1128/jvi.01310-25

**Published:** 2025-09-30

**Authors:** Ji Zhang, Yunhao Fan, Da An, Mingtian Mao, Zhanbao Guo, Jing Yang, Qiuyue Li, Siming Zhu, Guannan Li, Xin Chen, Zhengkui Zhou, Shuisheng Hou, Youxiang Diao, Yi Tang

**Affiliations:** 1 Institute of Animal Science of Chinese Academy of Agricultural Sciences, Hai’dian, Beijing, China; 2 College of Animal Science and Veterinary Medicine, Shandong Agricultural University, Tai'an, China; Wake Forest University School of Medicine, Winston-Salem, North Carolina, USA

**Keywords:** flavivirus, Tembusu virus, NS5, IFN-I signaling, NLS, karyopherin

## Abstract

Numerous species in the genus *Flavivirus* have been identified to pose serious threats to public health. Most of them have developed evasion strategies to counteract antiviral activity triggered by type I interferon (IFN-I), making prevention and control challenging. Our study reveals that Tembusu virus (TMUV), a *Flavivirus* that mainly causes severe disease in ducks, can inhibit IFN-I signaling via its NS5 protein, efficiently preventing the nuclear translocation of IFN-activated transcription factor STATs and significantly impairing transcriptional induction of IFN-stimulated genes. Interestingly, our research shows that 37–45 amino acid (aa) residues in the N-terminal region of TMUV-NS5 constitute a functional nuclear localization signal (*NLS*) that interacts with karyopherins, disrupting the nuclear transport system, in contrast to the classical *α/β NLS* found in most flaviviruses. Moreover, through in-depth analysis, we highlight that the nuclear trafficking competence of *NLS* is modulated by multiple determinants, such as the species and molecular size of cargo proteins, providing critical mechanistic insights into the differential subcellular localization of *Flavivirus* NS5. Altogether, our findings contribute to a better understanding of how TMUV-NS5 inhibits IFN-I signaling and offer novel perspectives on the evasion of the innate immune responses by *Flavivirus*.

**IMPORTANCE:**

Recent studies have demonstrated that various flaviviruses can inhibit the innate type I interferon (IFN-I) response. Similarly, Tembusu virus (TMUV), a highly epidemic *Flavivirus* among ducks, has been reported to inhibit IFN-I induction. In the present study, we confirm that TMUV is also an antagonist of IFN-I signaling, and its NS5 plays a key role. However, different from *α/β* nuclear localization signal (*NLS*) in most flaviviruses, 37–45 amino acid region in N-terminus of TMUV-NS5 has been identified as a crucial area for interaction with KPNAs, thus inhibiting nuclear transport of STATs. In addition, we further discovered that the nuclear localization activity of *NLS* is regulated by multiple factors, such as different sizes and types of the cargos, thereby leading to the distinct subcellular distribution of *Flavivirus* NS5.

## INTRODUCTION

Tembusu virus (TMUV), an enveloped, positive-sense, single-stranded RNA virus, is a contagious pathogen that mainly infects egg-laying ducks and has caused large economic losses in China since 2010 (1, 2). It belongs to the genus *Flavivirus,* and besides TMUV, the genus *Flavivirus* of the family Flaviviridae contains more than 70 members, including dengue virus (DENV), West Nile virus (WNV), Japanese encephalitis virus (JEV), yellow fever virus (YFV), and Zika virus (ZIKV) (3). Most of the viruses in this group are zoonotic pathogens that can cause serious human and animal diseases (4). To date, TMUV, which is primarily responsible for severe egg drop syndrome, has gradually expanded its host range beyond ducks, with chickens and geese also being susceptible to infection (5). Furthermore, a high serum-positive rate of TMUV antibodies has been detected among duck industry workers, indicating that the virus poses a potential threat to public health (6).

Flaviviruses have a genome of approximately 11 kb that encodes only one open reading frame (ORF). Cleavage by both host and viral proteases produces three structural proteins of core (C), membrane (prM), and envelope (E), followed by seven nonstructural (NS) proteins (NS1, NS2A, NS2B, NS3, NS4A, NS4B, and NS5) (7, 8). The C, prM, and E proteins are required for the formation of the viral particle, while the NS proteins are involved in establishing sites on membranes of the endoplasmic reticulum (termed viral replication complexes) where replication of viral RNA occurs (9). Among NS proteins, the *Flavivirus* NS5 protein is the largest and most conserved, and its N-terminus contains a methyltransferase (MTase) domain, while the C-terminus possesses an RNA-dependent RNA-polymerase (RdRp) activity (10).

Interferon (IFN)-mediated innate immunity, the first line of defense against invading viruses, plays a critical role in priming the adaptive immune response (11). One such system, type I IFN (IFN-I) (α/β), is the major effector cytokine in response to viral and other microbial infections. It regulates hundreds of gene products through the Janus kinase signal transducer and activation of transcription (JAK-STAT) signaling pathway (12, 13). After secretion, IFN-I first interacts with IFN α/β receptor (composed of two subunits, IFNAR1 and IFNAR2) on the cell surface, and subsequently, activated Janus kinase 1 (JAK1) and tyrosine kinase 2 (Tyk2) lead to the phosphorylation of signal transducers and activators of transcription (STAT1 and STAT2) at tyrosine residues (14). STATs, crucial players in the JAK-STAT signaling pathway, constitutively interact with the transcription factor IFN regulatory factor 9 (IRF9) to form a transcription factor complex known as IFN-stimulated gene factor 3 (ISGF3), serving as the key link between the cell surface and the nucleus (15). The nucleocytoplasmic transport process of such large proteins (usually over 40 kDa) always requires the participation of importing proteins karyopherin α or β (16, 17). After IFN stimulation, a specific member of the importing α family, importin α5 (KPNA1), recognizes a conditional nuclear localization signal (*NLS*) of pSTAT1 either in the form of ISGF3 or as a homodimer and then interacts with importin β1 (KPNB1), mediating the complex transmission into the nucleus (15, 18). Subsequently, these signals continue to induce the expression of ISGs that are responsible for both establishing antiviral states and disrupting various stages of the viral life cycle (19, 20).

To establish infection and replicate in the hosts, diverse flaviviruses have evolved powerful strategies to counteract both IFN-I induction and signaling, and their NS proteins consistently play pivotal roles in this inhibition (21–26). Recent studies determined that TMUV NS1 and NS2B3 proteins can inhibit the induction of IFN-β, indicating that it is an immunosuppressive virus similar to most flaviviruses (27–31). Notably, among the NS proteins, NS5 is known to be important in innate immune evasion, and its multiple roles have been identified in the antagonism of IFN-I signaling, including decreasing the expression or phosphorylation levels of STATs, reducing the formation of ISGF3, and suppressing the accumulation of STATs into the nucleus (32, 33). However, it remains to be determined whether TMUV also antagonizes IFN-I signaling and which protein(s) primarily mediate this suppression.

Here, we demonstrate that TMUV-NS5 protein is a major inhibitor of IFN-I signaling after infection and interacts with KPNAs via a functional *NLS_37–45_* in its N-terminus, thereby interfering with the nuclear accumulation of STATs. These findings provide new insights into the ability of TMUV to evade the innate immune response and identify a potential target region for its prevention and control.

## RESULTS

### TMUV antagonizes IFN-I signaling

**Fig 1:**
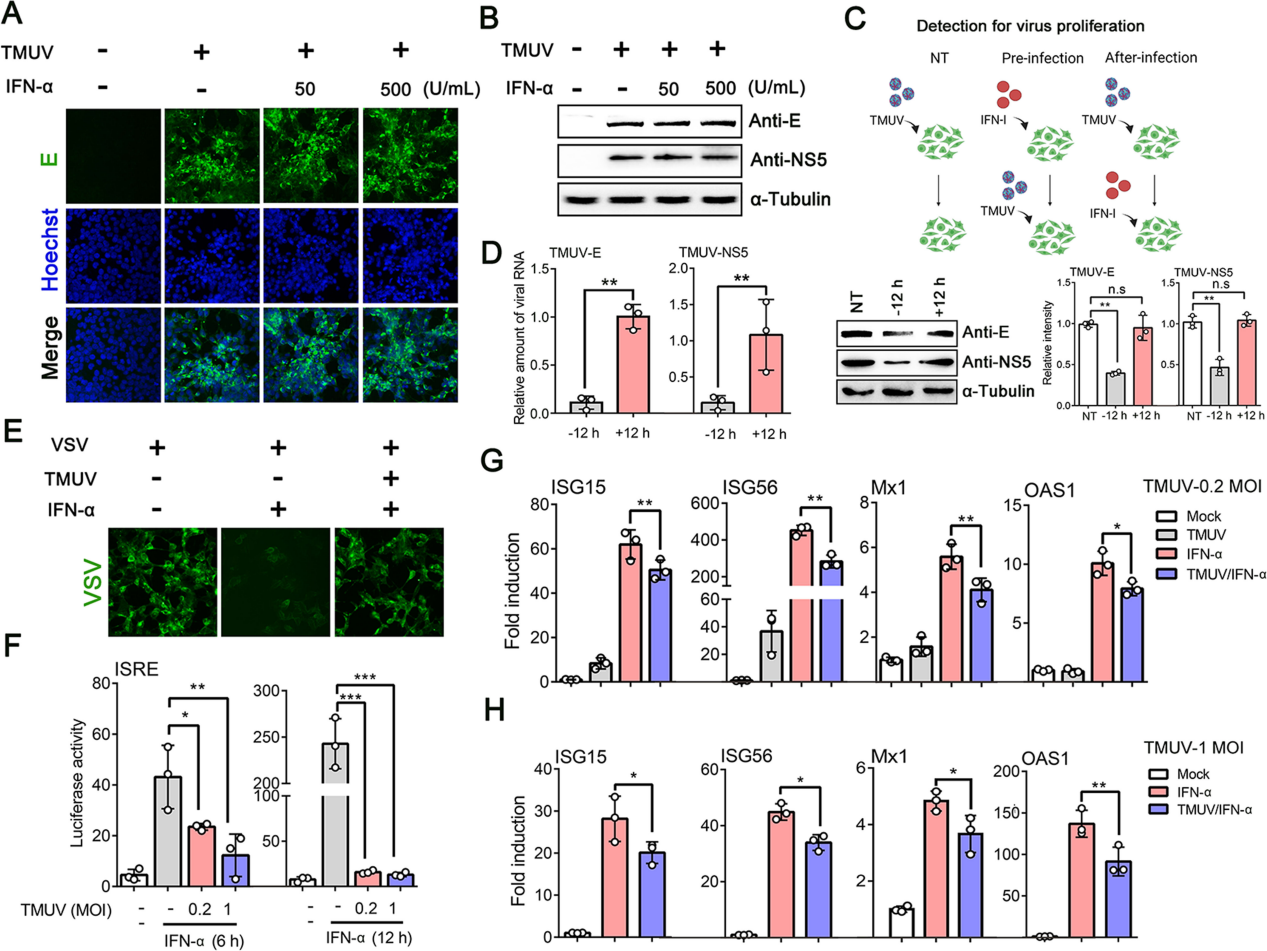
IFN-I signaling is suppressed by TMUV. (**A**) TMUV-infected (MOI = 1) HEK293 cells were stimulated with 50 U/ml and 500 U/mL of IFN-α for 12 h, and IFA was performed using TMUV-E antibody. (**B**) HEK293 cells were infected or not with TMUV (MOI = 1) for 24 h and then treated with IFN-α (50 U/mL, 500 U/mL) for 12 h. The virus proliferation was tested by WB with anti-E and NS5 antibodies. (**C, D**) HEK293 cells were infected with TMUV (MOI = 1), and these cells were either not treated with IFN-α (NT), pretreated with IFN-α (500 U/mL) for 12 h (−12 h), or treated with IFN-α (500 U/mL) 12 h (+12 h) after infection. The infection lasted for 24 h, and the virus proliferation was determined by WB (**C**) and qRT-PCR analysis (**D**). (**E**) TMUV-infected (MOI = 1) HEK293 cells were stimulated with 500 U/mL IFN-α for 12 h, then infected with VSV for 24 h. The VSV replication was monitored by IFA using fluorescence microscopy. (**F**) HEK293 cells were co-transfected with pISRE-TA-luc and pRL-SV40 for 24 h and subsequently infected with TMUV (MOI = 0.2, 1) for another 24 h. Measurements were taken after stimulation with IFN-α (500 U/mL) for 6 h or 12 h using a dual-luciferase reporter gene kit. (**G, H**) HEK293 cells infected with TMUV (MOI = 0.2 or 1) for 24 h and then incubated in the medium containing 500 U/mL IFN-α for 12 h or 24 h. The mRNA expression levels of representative ISGs, including ISG15, ISG56, Mx1, and OAS1, were measured by qRT-PCR. Density analysis of protein levels was quantified by ImageJ software. Data were presented as the means ± SEM, and comparisons between two groups were performed using an unpaired two-tailed Student’s t test. *P < 0.05, **P < 0.01, ***P < 0.001, and no significance (n.s). All experiments were independently repeated three times. IFN-α used for treating HEK293 cells is a human source.

The IFN-I system often defends against flavivirus infection effectively, but some pathogens have acquired specific mechanisms to limit this response (34). To examine the effects of IFN-I on TMUV, HEK293 cells were infected with TMUV (multiplicity of infection [MOI] = 1) for 24 h and then stimulated with IFN-α (50 and 500 U/mL) for 12 h. Using indirect immunofluorescence assay (IFA) and western blot (WB) analysis, we found that TMUV infection was not significantly affected by IFN-α treatment (Fig. 1A and B). We next confirmed that pre-infection treatment of these cells with IFN-α inhibited the replication of TMUV, but that the antiviral effect was less robust if it was added to the cells following infection (Fig. 1C and D). Additionally, mock- and TMUV-infected HEK293 cells were stimulated with IFN-α (500 U/mL) for 12 h and then inoculated with vesicular stomatitis virus (VSV) for 24 h. Here, IFA revealed that IFN-α could observably reduce VSV infection, but in TMUV-infected cells, its function was limited and VSV replication recovered (Fig. 1E). These results suggested that after TMUV infection, the antiviral effect of IFN-α is not exerted effectively (24, 35).

IFN binds to IFNARs and transmits signals through the JAK-STAT pathway, resulting in the activation of antiviral genes containing IFN-stimulated response element (ISRE) in their promoters (36). To further investigate the effects of TMUV on IFN-α-induced ISRE activation, HEK293 cells were co-transfected with a luciferase reporter construct (pISRE-TA-luc) and pRL-SV40, then infected with TMUV (MOI = 0.2, 1) for 24 h. Upon IFN-α treatment for 6 h, the ISRE promoter activity was about 45-fold higher than in the control cells (not with TMUV and IFN-α). However, in TMUV-infected cells, it had markedly lower levels, and when stimulated for 12 h, similar results were obtained (Fig. 1F). In addition, we also analyzed the mRNA expression of ISGs, the main executors for resisting viral infections (13). Following inoculation with TMUV (MOI = 0.2) and treatment with 500 U/mL of IFN-α for 12 or 24 h, the expression of representative ISGs (ISG15, ISG56, Mx1, and OAS1) was measured by qRT-PCR. After stimulation with IFN-α, the transcripts of ISGs increased 62-, 452-, 6-, and 10-fold (respectively) higher than normal controls (without both TMUV infection and IFN-α treatment), but these examined mRNA levels were reduced significantly after TMUV infection (Fig. 1G). Although these ISG levels were higher than those in viral infection alone, they were remarkably inhibited compared with mock-infected cells (with IFN-α treatment), which failed to effectively prevent further viral infection (37). When the infection dose increased (MOI = 1), the inhibition effects of TMUV on ISGs still existed (Fig. 1H). Altogether, these observations strongly suggested that TMUV could impair the immune response via decreasing ISG expression and, like other flaviviruses (such as DENV and LGTV), must encode the antagonist of IFN-I signaling (33).

### TMUV-NS5 is the main inhibitor for IFN-I signaling

**Fig 2:**
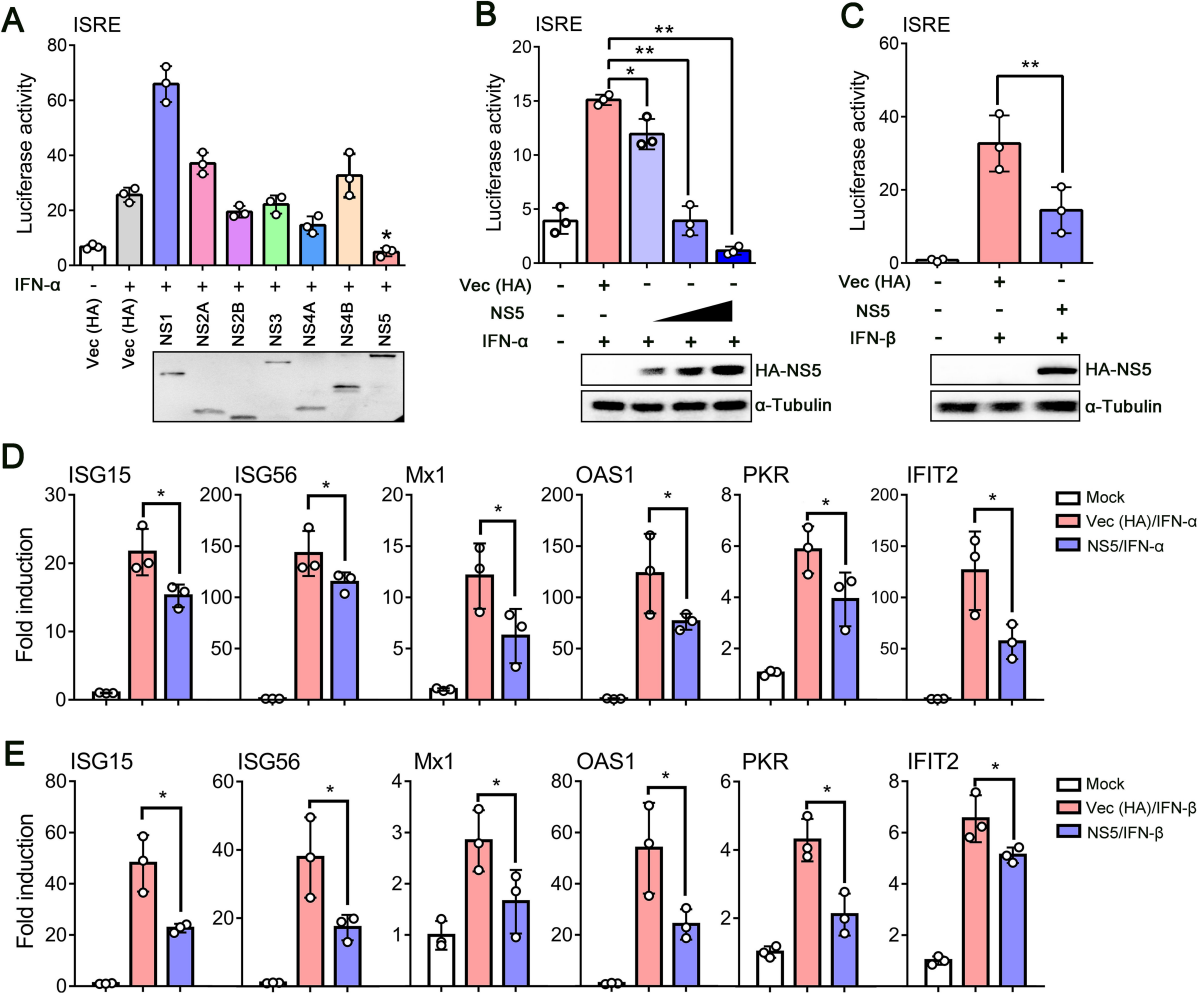
TMUV-NS5 is an inhibitor of IFN-I signaling. (**A**) HEK293 cells were co-transfected with empty pCAGGS-HA plasmids (as a control) or HA-tagged TMUV NS proteins (NS1, NS2A, NS2B, NS3, NS4A, NS4B, or NS5) with pISRE-TA-luc and pRL-SV40. Twenty-four hours later, the cells were stimulated with or without IFN-α (1,000 U/mL) for 6 h, then luciferase reporter assays were performed. (**B**) Increasing amounts of plasmid encoding NS5 were co-transfected into HEK293 cells with pISRE-TA-luc and pRL-SV40. An empty vector of pCAGGS-HA was used as a control. After 24 h, cells were untreated or treated with IFN-α (1,000 U/mL) for 6 h, and the results were analyzed by luciferase reporter assays. (**C**) HEK293 cells were co-transfected with pISRE-TA-luc, pRL-SV40 plasmids, HA-NS5 (with the empty vector of pCAGGS-HA as a control) for 24 h, subsequently stimulated with IFN-β (1,000 U/mL) for 12 h, and the reporter expression was examined using a dual-luciferase reporter assay kit. (**D, E**) HEK293 cells transfected with TMUV-NS5 or Vec (HA) as a control for 24 h and then treated with IFN-α (1,000 U/mL) (**D**), or -β (1,000 U/mL) (**E**) for 12 h or 24 h. The transcript levels of ISG15, ISG56, Mx1, OAS1, PKR, and IFIT2 were detected by qRT-PCR. Data were presented as the means ± SEM, and comparisons between two groups were performed using an unpaired two-tailed Student’s t test (*P < 0.05, **P < 0.01). All experiments were independently repeated three times. IFN-α or -β used for treating HEK293 cells is a human source.

To date, multiple flaviviruses have been demonstrated to inhibit the IFN-I signaling pathway, and NS proteins always play essential roles (38). Next, to determine which viral protein(s) antagonize IFN-I signaling, we generated plasmids individually encoding seven TMUV NS proteins fused to HA epitope tag and co-transfected HEK293 cells with each (along with pISRE-TA-luc and pRL-SV40). Upon IFN-α treatment for 12 h, the luciferase activity was measured by the dual-luciferase reporter assay. The results revealed that compared with negative controls (empty vector of pCAGGS-HA), ISRE promoter activity induced by IFN-α was significantly suppressed in NS5-expressing cells and decreased in a dose-dependent manner (Fig. 2A and B). The phenomenon was also observed in IFN-β-stimulated cells, and the mRNA levels of tested ISGs were significantly downregulated by TMUV-NS5 (Fig. 2C through E). These data indicated that the NS5 protein is the main inhibitor for IFN-I signaling after TMUV infection.

### ISGF3 heterotrimer formation is not inhibited by TMUV-NS5

**Fig 3:**
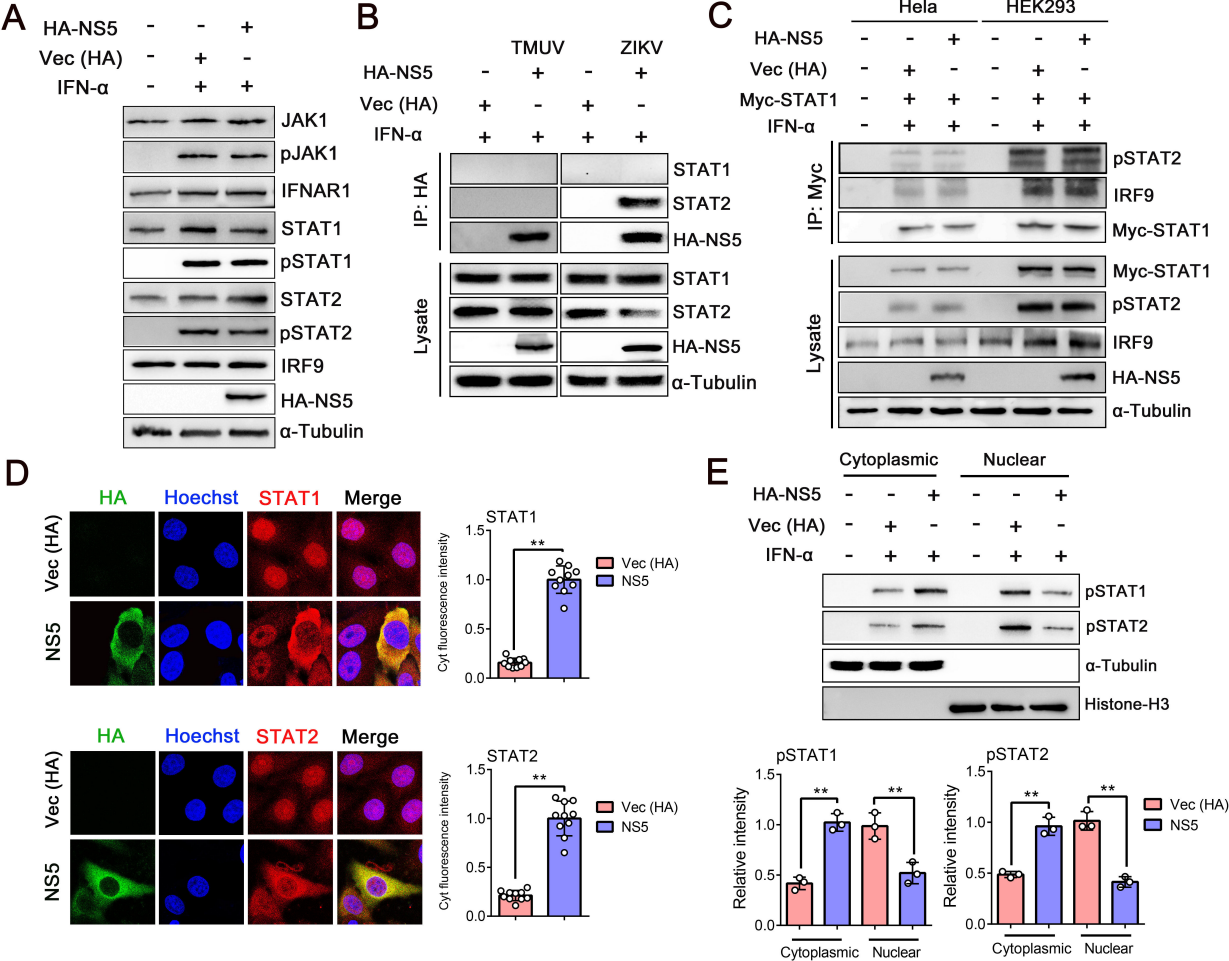
TMUV-NS5 inhibits the nuclear translocation of phosphorylated STATs. (**A**) HEK293 cells were transfected with the empty vector (Vec) or HA-NS5, and at 24 h post-transfection, cells were either left untreated or treated with 1,000 U/mL of IFN-α for 30 min and harvested for WB with antibodies against JAK1, pJAK1, IFNAR1, STAT1, pSTAT1, STAT2, pSTAT2, IRF9, or HA, as indicated. The same blot was incubated with α-Tubulin antibody as a protein loading control. (**B**) HEK293 cells were transfected with Vec (HA), TMUV-NS5, or ZIKV-NS5 for 24 h, and then the cells were stimulated with IFN-α (1,000 U/mL) for 30 min. Immunoprecipitation (IP) was performed with the HA-tag antibody, followed by IB with STAT1 and STAT2 antibodies, respectively. (**C**) HeLa or HEK293 cells were co-transfected Vec (HA) or HA-NS5 with Myc-STAT1 for 24 h. Then, cells were stimulated with IFN-α (1,000 U/mL) for 30 min, and IP was conducted with Myc-tag antibody, followed by IB with pSTAT2 and IRF9 antibody, respectively. (**D**) HeLa cells were transfected with Vec (HA) or HA-NS5 expression plasmid and were treated with IFN-α (1,000 U/mL) for 30 min. Then, cells were stained using STAT1, STAT2, and HA-tag antibodies. The fluorescence intensity in the cytoplasm of STATs was calculated by counting at least 10 cells using ImageJ software, and the cytoplasmic (Cyt) fluorescence intensity in the NS5-transfected group was set as the base value (1 assumed). (**E**) HEK293 cells were treated with 1,000 U/mL IFN-α in the presence or absence of NS5 protein. The component proteins were fractionated into nuclear and cytoplasmic fractions, and then the samples were subjected to WB with pSTAT1, pSTAT2, and HA-tag antibodies. Histone H3 and α-Tubulin were used as protein loading controls. Density analysis of protein levels was quantified by ImageJ software. Data were presented as the means ± SEM, and comparisons between two groups were performed using an unpaired two-tailed Student’s t test (**P < 0.01). All experiments were independently repeated three times. IFN-α used for treating HEK293 and HeLa cells is a human source.

IFN-I binding to IFNARs is followed by the phosphorylation of JAK1, Tyk2, STAT1, and STAT2 (36). To investigate whether these key players are directly affected by TMUV-NS5, HEK293 cells were transfected with HA-NS5 and then treated with IFN-α for 30 min. While the IFNAR1, JAK1, IRF9, STAT1, and STAT2 protein levels did not show a significant difference in the presence or absence of NS5 protein and after IFN-α treatment, the levels of phosphorylated JAK1 (pJAK1), pSTAT1, and pSTAT2 were greatly increased compared with mock-treated cells. However, no difference was observed with or without NS5 transfection (Fig. 3A). These results indicated that TMUV-NS5 does not decrease the protein levels of JAK1, IFNAR1, STATs, or their phosphorylation status after IFN-α stimulation.

In addition, the binding ability of TMUV-NS5 to STAT1 and STAT2 was examined. HEK293 cells were transfected with HA-tagged TMUV-NS5 or ZIKV-NS5 (as positive control) and subsequently treated with IFN-α (1,000 U/ml) for 30 min. Immunoprecipitation (IP) with HA-tag antibody, followed by immunoblotting (IB) with STAT1 and STAT2 antibodies, detected that ZIKV-NS5 could interact with STAT2, while no binding between TMUV-NS5 and any of the STATs (Fig. 3B).

When stimulated by IFN-I, phosphorylated STAT1 and STAT2 form a heterodimer and then associate with IRF9 to form the ISGF3 complex (39). To determine whether TMUV-NS5 affects the formation of ISGF3, we co-transfected both HeLa and HEK293 cells with Myc-STAT1 and HA-NS5 and then treated the cells with IFN-α (1,000 U/mL) for 30 min. IP with a Myc-tag antibody followed by IB with either pSTAT2 or IRF9 antibody indicated that no obvious difference was observed with or without NS5 transfection (Fig. 3C). These data suggested that the formation of ISGF3 is not remarkably affected by TMUV-NS5.

### TMUV-NS5 interrupts the nuclear accumulation of STATs

**Fig 4:**
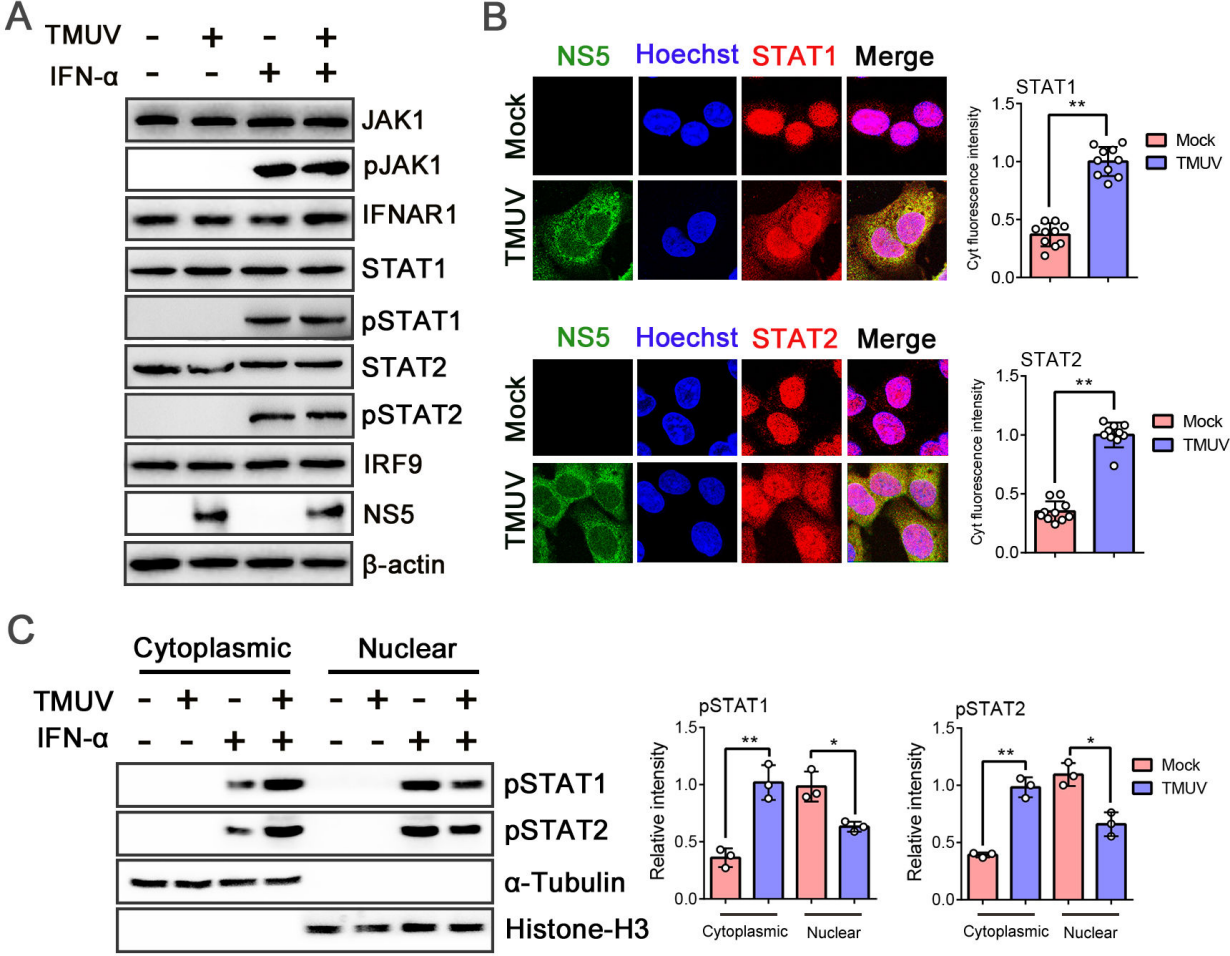
TMUV blocks the translocation of phosphorylated STATs into the nucleus. (**A**) HEK293 cells were infected with TMUV (MOI = 1) or not for 24 h and then treated with IFN-α (500 U/mL) for 30 min. Protein levels of JAK1, pJAK1, IFNAR1, STAT1, pSTAT1, STAT2, pSTAT2, IRF9, or TMUV-NS5 were determined by WB. (**B, C**) Mock- or TMUV- (MOI = 1) infected HEK293 cells were treated with IFN-α (500 U/mL) for 30 min. Then, these cells were fixed, and the nuclear translocation of STAT1 and STAT2 was monitored by IFA using confocal microscopy. The fluorescence intensity in the cytoplasm of STATs was calculated by counting at least 10 cells using ImageJ software, and the cytoplasmic (Cyt) fluorescence intensity in the TMUV-infected group was set as the base value (1 assumed) (**B**). Other whole-cell proteins were separated into nuclear and cytoplasmic fractions, and WB was performed using pSTAT1 and pSTAT2 antibodies. Histone-H3 and α-Tubulin were used as protein loading controls (**C**). Density analysis of protein levels was quantified by ImageJ software. Data were presented as the means ± SEM, and comparisons between two groups were performed using an unpaired two-tailed Student’s t test (*P < 0.05, **P < 0.01). All experiments were independently repeated three times. IFN-α used for treating HEK293 cells is a human source.

ISGF3 is the primary signal leading to the expression of target genes required for innate antiviral immunity. It enters into the nucleus and then initiates gene transcription by binding with ISRE (12). To examine whether the translocation step of STATs is inhibited by TMUV-NS5, we transfected HeLa cells with HA-NS5 and subsequently stimulated the cells with IFN-α (1,000 U/mL) for 30 min. Confocal microscopy revealed that, after IFN-α treatment, the majority of pSTAT1 and pSTAT2 translocated to the nucleus; however, they remained largely in the cytoplasm in NS5 expression cells (Fig. 3D). It was further confirmed by separating nuclear and cytoplasmic fractions of the cells after IFN-α treatment, and phosphorylated STATs in these two fractions were detected by WB. After IFN-α stimulation, more pSTAT1 and pSTAT2 were found in the nuclear fraction than in the cytoplasm. In contrast, in the presence of TMUV-NS5, the nuclear pSTAT levels were reduced and largely remained in the cytoplasm (Fig. 3E). Furthermore, we investigated the effect of TMUV infection on the IFN-I signaling pathway. HEK293 cells were either infected with TMUV (MOI = 1) or mock-infected for 24 h, followed by treatment with IFN-α (500 U/mL) for 30 min. As shown in Fig. 4, TMUV had no impact on the levels or phosphorylation status of key transcription factors, whereas the translocation process of pSTATs into the nucleus was significantly inhibited. However, this inhibition appeared to be less pronounced than the effect of NS5 transfected alone. The underlying reason may mostly lie in the fact that viral infection often initiates a cascade of highly complex processes, and specifically, the activation of IFN-I mediated by certain viral components, including ssRNA and dsRNA, can partially counteract the antagonistic effects of NS5. Together, these data indicated that TMUV-NS5 inhibits STATs nuclear accumulation after IFN-α treatment.

### TMUV-NS5 interacts with KPNAs and inhibits STAT1 nuclear transfer

**Fig 5:**
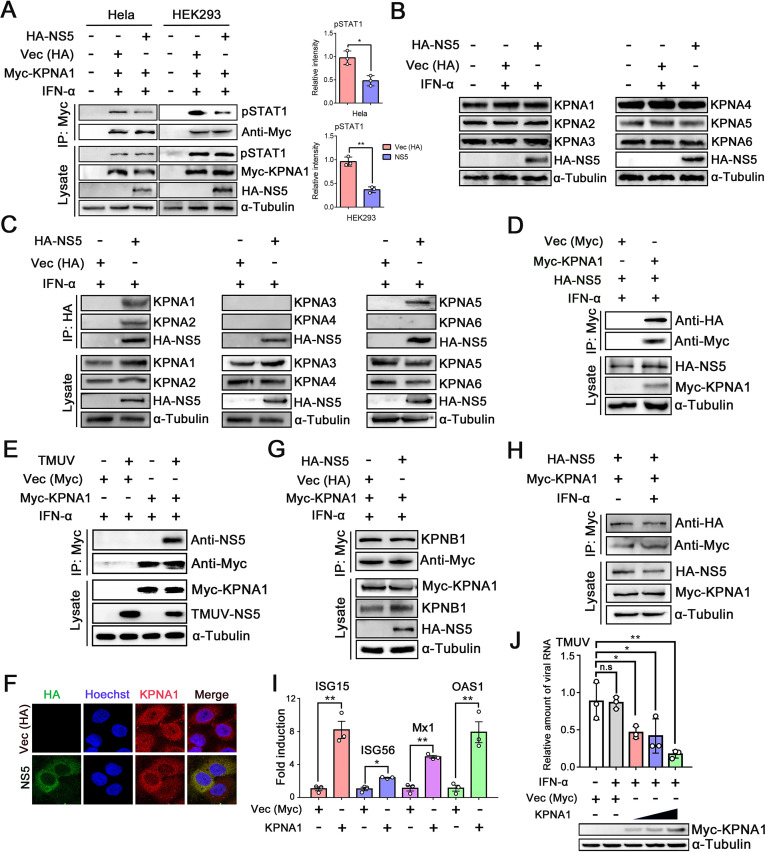
TMUV-NS5 interacts with KPNAs, resulting in the reduction of pSTAT1 nuclear traffic. (**A**) HeLa and HEK293 cells were co-transfected with the indicated plasmids of Vec (HA), HA-NS5, and Myc-KPNA1. At 24 h post-transfection, cells were treated with 1,000 U/mL IFN-α for 30 min, and then IP was performed with an anti-Myc antibody and subsequently IB with the pSTAT1 antibody. (**B**) HeLa cells were transfected with the HA-NS5 plasmid or Vec (HA). Twenty-four hours after transfection, cells were stimulated with IFN-α (1,000 U/mL) for 30 min, and the expression levels of endogenous KPNA1, KPNA2, KPNA3, KPNA4, KPNA5, and KPNA6 were analyzed by WB. (**C**) HeLa cells were treated with IFN-α (1,000 U/mL) for 30 min in the presence or absence of HA-NS5, and IP was conducted with anti-HA antibodies, subsequently IB with KPNA1, KPNA2, KPNA3, KPNA4, KPNA5, and KPNA6 antibodies. An empty vector of pCAGGS-HA was used as a control. (**D**) HA-NS5 and Myc-KPNA1 (with the empty vector of pCMV-Myc as a control) were co-transfected into HEK293 cells for 24 h and then treated with IFN-α (1,000 U/mL) for 30 min. IP was conducted with an anti-Myc antibody and subsequently IB with an HA-tag antibody. (**E**) HEK293 cells were first transfected with the empty vector (pCMV-Myc) or Myc-KPNA1 for 24 h and then infected with TMUV (MOI = 5) or not for another 24 h. After being treated with IFN-α (500 U/mL) for 30 min, co-IP analysis was performed with anti-Myc antibody, subsequently IB with TMUV-NS5 antibody. (**F**) HeLa cells were transfected with Vec (HA) or HA-NS5 plasmids, and at 24 h post-transfection, cells were treated with 1,000 U/mL IFN-α for 30 min. Then, IFA was performed with anti-HA and KPNA1 antibodies. (**G**) Vec (HA) or HA-NS5 were co-transfected with Myc-KPNA1 into HEK293 cells, and at 24 h post-transfection, cells were treated with IFN-α (1,000 U/mL) for 30 min. IP was performed with an anti-Myc antibody and subsequently IB with KPNB1 antibody. (**H**) HEK293 cells, which were co-transfected with HA-NS5 and Myc-KPNA1 for 24 h, were untreated and treated with IFN-α (1,000 U/mL) for 30 min, and IP was conducted with anti-Myc antibody, subsequently IB with HA-tag antibody. (**I**) HEK293 cells were transfected with Myc-KPNA1 or Vec (Myc) as a control, then infected with TMUV (MOI = 1) for 24 h. After treating the cells with IFN-α (500 U/mL) for 6 h, the ISGs (ISG15, ISG56, Mx1, OAS1) mRNA expressions were measured by qRT-PCR. (**J**) After transfection with different doses of Myc-KPNA1 plasmid, HEK293 cells were inoculated with TMUV (MOI = 1) for another 24 h. Then, the cells were treated with IFN-α (500 U/ml) for 6 h, and TMUV replication levels were detected by qRT-PCR. Density analysis of protein levels was quantified by ImageJ software. Data were presented as the means ± SEM, and comparisons between two groups were performed using an unpaired two-tailed Student’s t test. *P < 0.05, **P < 0.01 and no significance (n.s). All experiments were independently repeated three times. IFN-α used for treating HEK293 and HeLa cells is a human source.

After stimulation, pSTAT1 accumulates in the nucleus mainly with the assistance of KPNA1, which functions as the specific NLS receptor (15, 18). Subsequently, we investigated whether TMUV-NS5 interrupts the interaction between STAT1 and KPNA1. HeLa and HEK293 cells were co-transfected with plasmids encoding HA-NS5 and Myc-KPNA1. An empty vector (pCAGGS-HA) was used as a control. IFN-α was added 24 h after transfection, and the cells were then harvested for co-IP (co-IP) assay. The results revealed that in TMUV-NS5-transfected cells, the coprecipitation of pSTAT1 with KPNA1 was significantly lower than that in controls, suggesting that NS5 protein acts as an inhibitor of pSTAT1-KPNA1 binding (Fig. 5A).

Next, the protein levels of KPNAs were determined for cells transfected with the HA-NS5 plasmid or an empty vector (pCAGGS-HA) as a control. Here, regardless of the presence or absence of NS5 protein, there was no significant influence on the levels of KPNAs (KPNA1, KPNA2, KPNA3, KPNA4, KPNA5, and KPNA6) (Fig. 5B). In summary, TMUV-NS5 inhibits the interaction of pSTAT1 with KPNA1 but does not alter the levels of KPNA1 or other KPNAs.

Given the ability of TMUV-NS5 to prevent the pSTAT1-KPNA1 binding but not reduce protein levels of KPNAs, we question whether it might interact with KPNA1 or other NLS receptors. Accordingly, HeLa cells were transfected with HA-NS5 or Vec (HA) and stimulated with IFN-α. Co-IP was performed with an anti-HA antibody, and the precipitated material was analyzed by IB with antibodies recognizing KPNA1~6. The results revealed that TMUV-NS5 specifically interacted with KPNA1, KPNA2, and KPNA5 (Fig. 5C). Next, to confirm the interaction of TMUV-NS5 with KPNA1, HEK293 cells were co-expressed with HA-NS5 and Myc-tagged KPNA1 (an empty vector of pCMV-Myc was used as a control). Upon IFN-α treatment, the result of IP with anti-Myc and subsequent IB with anti-HA revealed that TMUV-NS5 coprecipitated with KPNA1 (Fig. 5D). Subsequently, we transfected empty vector (pCMV-Myc) or Myc-KPNA1 into HEK293 cells and then infected with TMUV (MOI = 5) or not for another 24 h. After being treated with IFN-α (500 U/mL) for 30 min, we could detect the interaction between KPNA1 and NS5 when co-IP analysis was performed with anti-Myc antibody, subsequently IB with TMUV-NS5 antibody (Fig. 5E). Furthermore, IFA demonstrated that NS5 and KPNA1 were co-localized in the cytoplasm, and NS5 hindered the migration of KPNA1 into the nucleus (Fig. 5F).

KPNAs recognize and bind cargo proteins in the cytoplasm, linking them to KPNB through the importin β-binding domain, and then mediate the interaction of the trimeric complex with the nuclear pore (17). To determine the binding activity of KPNA1 with KPNB1 in NS5-expressing cells, we co-transfected HA-NS5 and Myc-KPNA1 into HEK293 cells. IP with anti-Myc and then IB with anti-KPNB1 showed that KPNA1-KPNB1-binding activity was not influenced by TMUV-NS5 (Fig. 5G). Furthermore, to detect whether IFN-α is necessary for TMUV-NS5 binding to KPNA1, co-transfected HA-NS5 and Myc-KPNA1 into HEK293 cells were stimulated with and without IFN-α. Co-IP results showed that without IFN-α treatment, TMUV-NS5 could also interact with KPNA1 (Fig. 5H). These data suggested that the interaction of TMUV-NS5 with KPNA1 partially inhibits the pSTAT1-KPNA1 association, thus explaining how the NS5 protein may suppress pSTAT1 nuclear accumulation.

Nuclear import machinery plays a major role in the immune system (40). To analyze the effect of KPNA1 on the antiviral status of the host, we transfected Myc-KPNA1 in HEK293 cells (with the empty vector of pCMV-Myc as a control), followed by TMUV infection (MOI = 1) and then treated these cells with IFN-α (500 U/mL). The results demonstrated that compared with control cells, KPNA1 overexpression dramatically increased the mRNA levels of ISGs, and the antiviral effects of IFN-I were restored (Fig. 5I and J). Thus, we concluded that the nuclear transport system plays a positive role in the antiviral response against TMUV infection.

### The *NLS_37–45_* region in the N-terminus is critical for antagonizing IFN-I signaling

**Fig 6:**
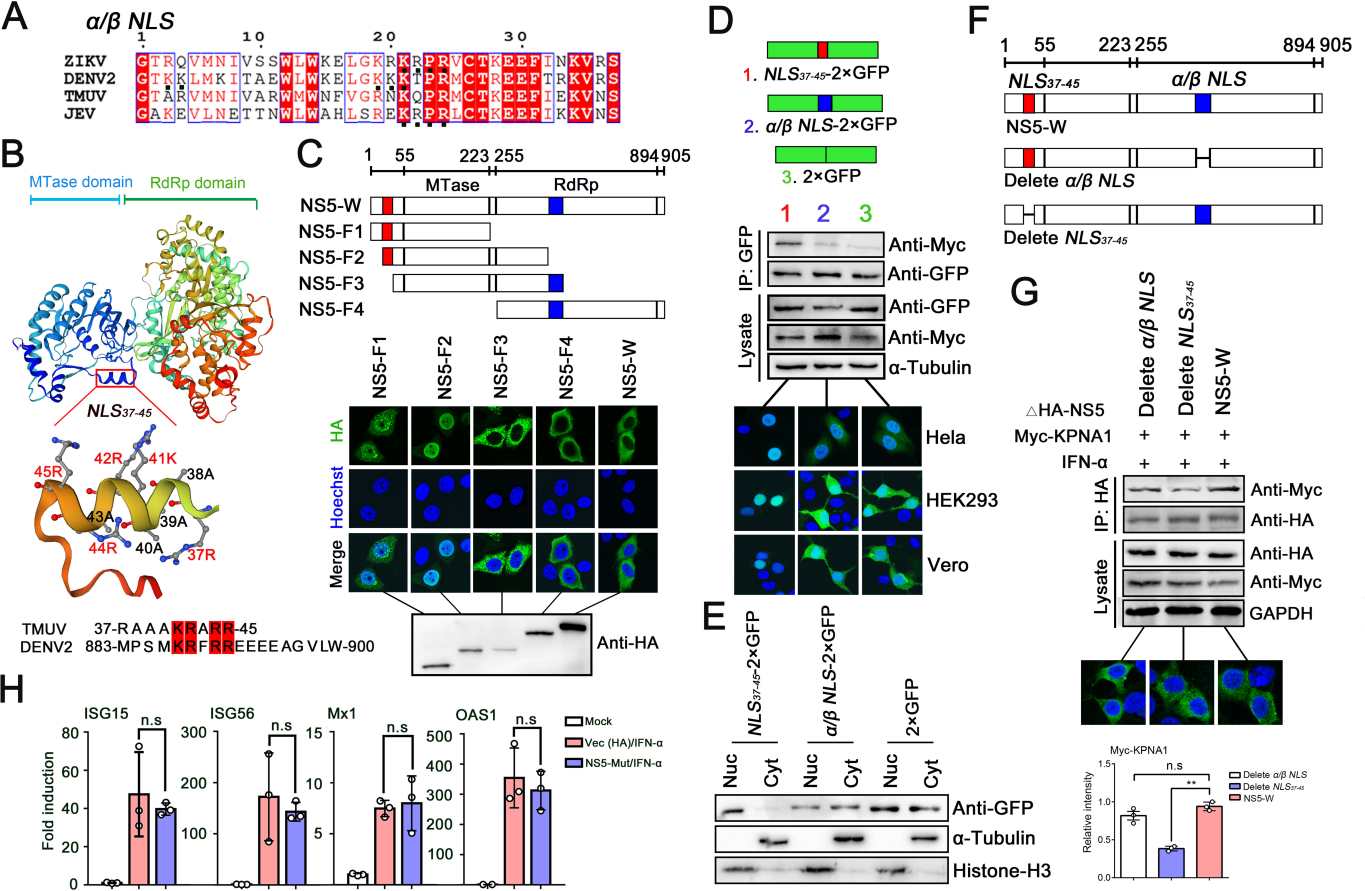
The *NLS_37–45_* region is identified as the main region for TMUV-NS5 interaction with KPNAs. (**A**) Sequence variation of TMUV *α/β NLS* was aligned with DENV2, ZIKV, and JEV (GenBank Accession No. ABW82013, AMO03410, and AAA21436) using DNASTAR software. (**B**) TMUV-NS5 amino acid sequence was analyzed by two software (PSORT II and cNLS Mapper), and *NLS_37–45_*, which is marked in the TMUV-NS5 structure (analyzed by SWISS-MODEL), was identified as the potential region recognized by KPNAs. In addition, *NLS_37–45_* was aligned with DENV2 *NLS* identified in Cter18 residues (GenBank Accession No. ABW82013). (**C**) TMUV-NS5 was truncated into F1 (1–223 aa), F2 (37–370 aa), F3 (46–407 aa), and F4 (255–905 aa) fragments and transfected into HeLa cells, respectively. Subsequently, IFA was performed to detect the cellular location. (**D**) 2 × GFP, *NLS_37–45_*−2 × GFP or *α/β NLS* 2 × GFP plasmids were co-transfected with Myc-KPNA1 into HEK293 cells for co-IP tests and HeLa, HEK293, Vero cells for IFA. (**E**) HEK293 cells were transfected with *NLS_37–45_* fused 2 × GFP, *α/β NLS*-2 ×GFP, and 2 × GFP plasmids. Then, the component proteins were fractionated into nuclear and cytoplasmic fractions, and samples were subjected to WB with a GFP-tag antibody. Histone H3 and α-Tubulin were used as protein loading controls. (**F, G**) TMUV-NS5 deletion mutants were constructed on the basis of the original plasmid (**F**), and then the two NS5 mutant plasmids were respectively transfected into HEK293 cells with Myc-KPNA1 for 24 h. After stimulation with IFN-α (1,000 U/mL) for 30 min, co-IP tests and IFA were performed (**G**). (**H**) An empty vector of pCAGGS-HA (Vec) and mutant NS5 plasmids, which deleted functional *NLS_37–45_* (NS5-Mut), were, respectively, transfected into HEK293 cells for 24 h. After stimulation with IFN-α (1,000 U/mL) for 12 or 24 h, the ISGs mRNA expression levels were analyzed by qRT-PCR. Density analysis of protein levels was quantified by ImageJ software. Data were presented as the means ± SEM, and comparisons between two groups were performed using an unpaired two-tailed Student’s t test. **P < 0.01, no significance (n.s). All experiments were independently repeated three times. IFN-α used for treating HEK293 cells is a human source.

*NLS*, which consists of either one (monopartite) or two (bipartite) stretches of basic aa, is the region recognized by KPNAs (17). Among various flaviviruses, NS5, a classical *α/β NLS* in the RdRp domain, is responsible for its interaction with KPNAs, and nuclear accumulation of NS5 mainly relies on this transport process (41, 42). However, the *α/β NLS* sequence in TMUV was found to be not conserved when aligning with the corresponding region such as DENV2/ZIKV-NS5 (located in the nucleus) and JEV-NS5 (located in the cytoplasm) (Fig. 6A). Accordingly, using PSORT II and cNLS Mapper software, we found that a potential region (aa at 37–45 positions) in the N-terminus of TMUV-NS5 has a typical structure of Class 2 monopartite *NLS* and the basic aa were identical in composition to the functional *NLS* area identified in DENV2 C-terminal 18 residues (Cter_18_) (Fig. 6B) (43, 44). Based on the possible *NLS* region predicted above, four truncations of F1 (1–223 aa), F2 (37–370 aa) which include the *NLS_37–45_* region and F3 (46–407 aa), F4 (255–905 aa) which include the *α/β NLS* region were cloned into the pCAGGS vector, and then transfected into HeLa cells. Via IFA, we found that NS5-F1 and F2 truncations were detected in the nucleus, while NS5-F3 and F4 were detected in the cytoplasm (Fig. 6C). In addition, the ability of *NLS* to carry GFP into the nucleus is an important indicator for evaluating its function (17, 41). Next, we also fused *NLS_37–45_* and *α/β NLS* of TMUV-NS5 with 2 × GFP and transfected them with Myc-KPNA1 into HEK293, HeLa, and Vero cells, respectively. As shown in Fig. 6D and E, the *NLS_37–45_* region had a strong interaction with KPNA1 and was sufficient to drive 2 × GFP completely into the nucleus. These findings suggested that *NLS_37–45_* in the N-terminus is the functional *NLS*, but not *α/β NLS* region in TMUV-NS5.

Lastly, we constructed two NS5 mutations, deleting either *α/β NLS* or *NLS_37–45_*, and transfected them with Myc-KPNA1 into HEK293 cells (Fig. 6F). After 24 h, by IFA, we found that both mutants were localized in the cytoplasm, which was the same as that of the wild-type NS5. However, co-IP assays showed that, after *NLS_37–45_* deletion, the interaction between NS5 and KPNA1 was weakened significantly (Fig. 6G). Additionally, we analyzed the effects of *NLS_37–45_* absence on the expression of ISGs. The NS5 mutation, which deleted the *NLS_37–45_* region (NS5-Mut), or empty vector of pCAGGS-HA was transfected into HEK293 cells for 24 h and stimulated with IFN-α (1,000 U/mL) for 12 h or 24 h. The qRT-PCR analysis revealed that after deletion of *NLS_37–45_*, NS5-Mut abolished the capacity of significantly inhibiting ISGs expression (Fig. 6H). These results confirmed that *NLS_37–45_* is the critical region responsible for TMUV-NS5 interaction with KPNAs and the inhibition of IFN-I signaling.

### Further analysis of the functional TMUV-NS5 *NLS_37–45_* region

**Fig 7:**
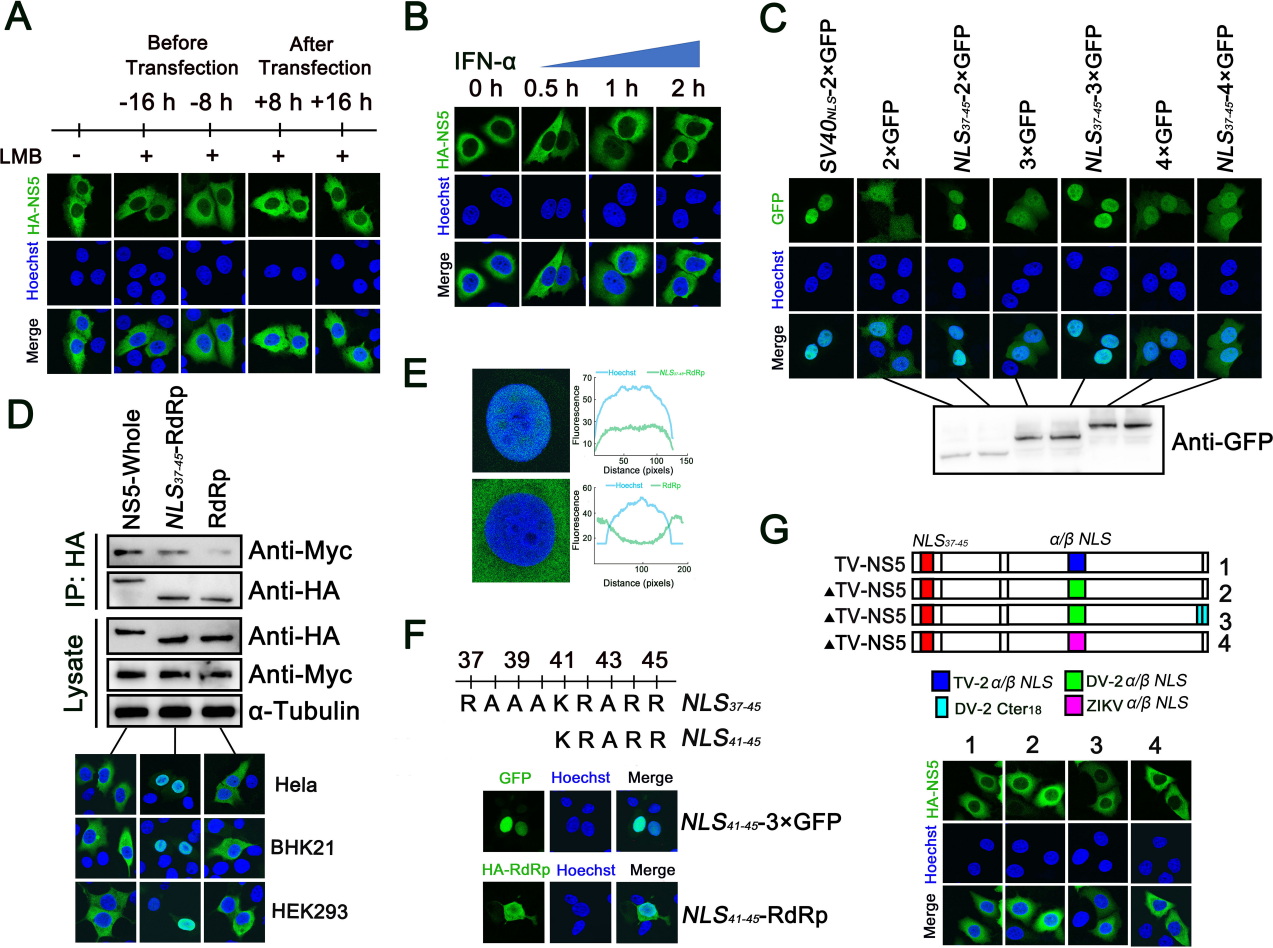
Further analysis of functional *NLS_37–45_*. (**A**) HeLa cells were handled with LMB (20 nM) for 8 h or 16 h before or after HA-NS5 transfection, and cellular localization of NS5 protein was detected by IFA. (**B**) HA-NS5 was transfected into HeLa cells for 24 h and then stimulated with IFN-α (1,000 U/mL) for 0.5, 1, or 2 h, respectively. IFA was performed with an HA-tag antibody. (**C**) *SV40_NLS_*-2 × GFP, 2 × GFP, 3 × GFP, 4 × GFP or *NLS_37–45_* fused 2 × GFP, 3 × GFP, 4 × GFP plasmids were transfected into HeLa cells, respectively, for 24 h, and then IFA was performed. (**D**) HA-tag fused TMUV-NS5, *NLS_37–45_*-RdRp, and RdRp plasmids were transfected into HeLa, BHK21, or HEK293 cells, respectively, for 24 h, and IFA was performed to detect their localization. HEK293 cells were co-transfected with Myc-KPNA1 and the plasmids above, and after 24 h post-transfection, a co-IP assay was performed with whole-cell lysates by using anti-HA antibody and subsequently IB with Myc-tag antibody. (**E**) TMUV *NLS_37–45_* fused RdRp and RdRp domain were transfected into HeLa cells for 24 h, and confocal microscopy was used to identify the localization. The line-scan graph showing the fluorescence intensities was measured by ImageJ software. (**F**) The *NLS_37–45_* truncation (*NLS_41–45_*) was fused with 3 × GFP and TMUV-RdRp domain plasmids. Then, they were transfected into HEK293 cells, respectively, for 24 h, and IFA was performed to detect the cellular localization. (**G**) The *α/β NLS* from ZIKV (GenBank Accession No. AMO03410) or both *α/β NLS* and Cter_18_ residues from DENV2 (GenBank Accession No. ABW82013) substitutions of mutant TMUV-NS5 plasmids were transfected into HeLa cells for 24 h, and their cellular localizations were analyzed by IFA. All experiments were independently repeated three times. IFN-α used for treating HeLa cells is human source.

*Flavivirus* NS5 protein has different nuclear localization, and it was reported that NS5 of DENV2 and ZIKV accumulated in the nucleus, while JEV-NS5 and DENV4-NS5 were found in the cytoplasm (41, 42, 45). In the present study, we observed that TMUV-NS5 is located in the cytoplasm, as previously reported (46). Next, to verify whether TMUV-NS5 shuttles between the cytoplasm and nucleus, leptomycin B (LMB), the nuclear export inhibitor, was used to treat HeLa cells before and after transfection for 8 or 16 h. We found that the NS5 protein still did not appear in the nucleus (Fig. 7A). In addition, to determine whether IFN-I stimulation changes the localization of TMUV-NS5, HeLa cells were transfected with HA-NS5 for 24 h and then treated with IFN-α (1,000 U/mL). IFA results showed that treatment of IFN-α did not change its location (Fig. 7B).

We have identified the interaction between TMUV-NS5 and KPNAs, but NS5 is localized in the cytoplasm. This phenomenon aroused our interest, and according to the above data (NS5 truncations of F1 and F2 fragments are located in the nucleus), we speculated that it may be related to the size of cargo proteins. Therefore, to further analyze this point, *NLS_37–45_*−2 × GFP, *NLS_37–45_*−3 × GFP (which has a similar molecular weight to the RdRp domain), and *NLS_37–45_*−4 × GFP (which has a similar molecular weight to the NS5 protein) expression vectors were constructed. Subsequently, these vectors were transfected into HeLa cells for 24 h. IFA analysis showed that, except for 4 × GFP, both 2 × GFP and 3 × GFP could be completely located in the nucleus with the association of *NLS_37–45_*, indicating that the carrying capacity of functional *NLS* is closely related to the size of cargo proteins (Fig. 7C). Similarly, the RdRp domain, which is originally localized in the cytoplasm, translocated into the nucleus when *NLS_37–45_* was added to its N-terminus and exhibited strong interaction with KPNA1 (Fig. 7D and E).

Interestingly, DENV2-NS5, which has a typical *NLS* in the Cter_18_, is located in the nucleus, but TMUV-NS5 is seen in the cytoplasm, although *NLS_37–45_* has the same composition of basic aa (Fig. 6B) (43). This indicates that the molecular weight is not the only factor affecting cargo proteins’ entry into the nucleus. Thus, we next constructed *NLS_41–45_* (truncated *NLS_37–45_*), which retained Class 2 *NLS* structure, fused 3 × GFP or RdRP domain plasmids. As shown in Fig. 7F, *NLS_41–45_*−3 × GFP was completely located in the nucleus, but for *NLS_41–45_*-RdRP (which has a similar molecular weight to 3 × GFP), it was dispersedly expressed in the cell. When DENV2 functional *α/β NLS* and Cter_18_ were replaced in TMUV-NS5, confocal microscopy still revealed that these substitutions did not change its subcellular localization, and this phenomenon was also seen in ZIKV-NS5 *α/β NLS* substitution (Fig. 7G). Based on the above data, we conclude that although *NLS* serves as the key region for KPNAs combining with cargo proteins, the cellular localization of these proteins is regulated by multiple factors, including but not limited to their size and type.

## DISCUSSION

**Fig 8:**
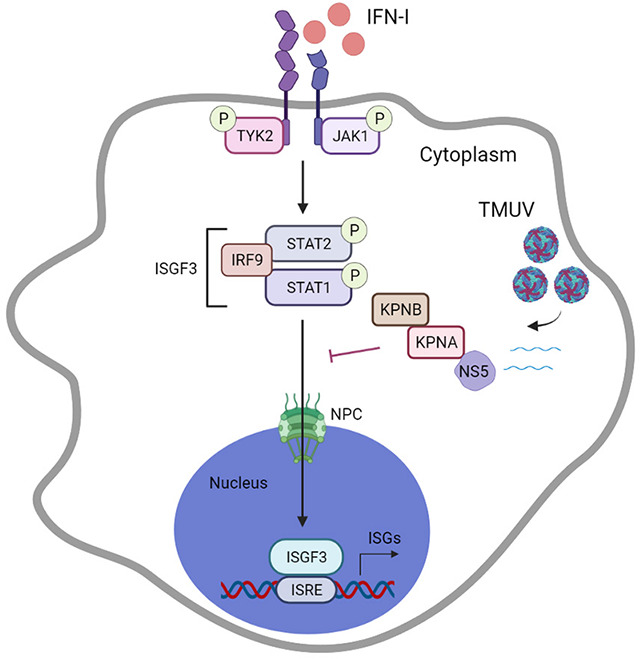
TMUV-NS5 antagonizes IFN-I signaling. Upon stimulation with IFN-I, phosphorylated STATs associate with IRF9 to form the ISGF3 complex, which is then translocated to the nucleus with the assistance of the nuclear transport system. This process leads to the activation of ISRE and subsequently induces the production of antiviral ISGs. TMUV-NS5 protein targets KPNAs (primarily through 37–45 aa in the N-terminus), thereby inhibiting activated STATs nuclear translocation and subverting IFN-I signaling.

As the first line of defense against viral infection, the innate immunity induces cells to enter an antiviral state, thereby inhibiting viral spread and replication. However, to establish successful infection, flaviviruses have evolved diverse strategies to evade the host antiviral response, which is primarily mediated by IFN-I, and the NS proteins play an important role (34, 47, 48). In this study, we demonstrated that TMUV (of the genus *Flavivirus*) can block IFN-I signaling, and its NS5 protein is confirmed as the direct and potent viral inhibitor of the JAK-STAT pathway (Fig. 8). Certainly, beyond NS5, we also discovered that subgenomic flavivirus RNA (sfRNA) of TMUV exerts a significant inhibitory effect on both IFN-I production and even its subsequent signaling cascade (data not shown), consistent with reports in other flaviviruses (49, 50). Currently, our research focuses on the role of NS5, while investigations on sfRNA are still in progress.

The mechanisms of immune evasion have demonstrated that LGTV-NS5 suppresses STAT1 phosphorylation in response to IFN stimulation, while JEV-NS5 inhibits STAT1 nuclear translocation, as well as the tyrosine phosphorylation of Tyk2 and STAT1 (24, 51). In addition, WNV-NS5 prevents phosphorylated STAT1 accumulation and blocks IFN-dependent gene expression (25). In contrast, our results identified that in the presence of TMUV-NS5, protein levels or the phosphorylation status of key transcription factors (IFNAR, JAK, and STATs) are not affected, and the ISGF3 heterotrimer formation is not altered. Moreover, unlike DENV, ZIKV, and YFV, TMUV-NS5 does not mediate STATs binding and degradation (23, 52, 53). Instead, when analyzing the nuclear localization of the ISGF3 complex, we found that IFN-I activated STAT1/2 fails to accumulate in the nucleus efficiently. Further investigation revealed that TMUV-NS5 antagonizes the nuclear translocation process of phosphorylated STATs and partially impairs the nuclear transport machinery. These data provide evidence that TMUV-NS5 acts as a major inhibitor of IFN-I signaling and that the involved strategy warrants further exploration.

After being stimulated by IFN-I, STAT1 is activated by tyrosine phosphorylation at amino acid 701 and then primarily interacts with KPNA1 (importin α5) via an exposed *NLS* on the surface of tyrosine phosphorylated STAT1 (pSTAT1), thereby facilitating the nuclear translocation of the ISGF3 complex (18, 54). In the present study, we demonstrated that the reduction of STATs nuclear translocation is not related to their expression, phosphorylation, or ISGF3 formation. Of note, the interaction between pSTAT1 and KPNA1 was significantly impaired in NS5-expressing cells. This phenomenon has been observed in 3C^pro^ of the foot-and-mouth disease virus (FMDV) or Nsp1β of the porcine reproductive and respiratory syndrome virus (PRRSV) (55, 56); however, the key distinction is that no degradation was found in KPNA1, even the protein levels of other KPNAs (KPNA2~6). Furthermore, the antagonistic function of STAT1 trafficking by some specific viral proteins results from its competitive association with KPNAs, including VP24 of the Ebola virus (EBOV), hepatitis B virus (HBV) polymerase interaction with KPNA1, and the ORF6 protein of the severe acute respiratory syndrome coronavirus (SARS-CoV) tethering KPNA2 (57–59). Consistent with the previously described mechanism antagonizing the KPNA1-pSTAT1 interaction, our findings indicated that TMUV-NS5 exhibits a similar capacity to bind with KPNAs (1, 2, and 5), thus blocking the translocation process of main factors into the nucleus.

KPNAs recognize the *NLS* region which enriches the basic aa in cargo proteins via armadillo (ARM) motifs and interacts with them (17). Among multiple flaviviruses, a basic-residue-rich area in the classical 37-amino-acid interdomain (*α/β NLS*) of the RdRp domain has been confirmed to mediate interactions between various NS5 proteins and KPNAs (including DENV, JEV, and ZIKV) (41, 42, 60, 61). However, TMUV-NS5 *α/β NLS* lacks a classical enrichment of basic aa, indicating that it may not perform a similar function (46). Accordingly, we analyzed the TMUV-NS5 sequence using the software packages PSORT II (62) and cNLS Mapper (63), predicting a functional *NLS* region at 37–45 aa in the N-terminus (44). This *NLS_37–45_* region exhibits a typical structure of Class 2 *NLS* and shares the same key basic aa composition as the newly identified *NLS* in DENV2 (43). Notably, in-depth analysis confirmed that *NLS_37–45_* displays a strong nuclear localization activity but not *α/β NLS*, and deletion of *NLS_37–45_* significantly impairs the binding of NS5 with KPNA1. More importantly, *NLS_37–45_* deficiency leads to NS5 no longer exerting the inhibitory effects on ISG production, highlighting that 37–45 aa is the main region that mediates interactions with KPNAs and is primarily responsible for TMUV-NS5 antagonizing IFN-I signaling.

Intriguingly, although TMUV-NS5 interacts with KPNAs, we still did not detect its nuclear localization (46), different from other *Flavivirus* NS5 that interacts with KPNAs and assists their nuclear localization (42, 61). In subsequent studies, we found that the *NLS_37–45_* region of TMUV-NS5 can carry various NS5 truncations, a series of GFP polyprotein and RdRp domain to the nucleus. However, 4 × GFP, which has a similar molecular weight to the full-length NS5, could not be transported to the nucleus with the assistance of *NLS_37–45_*. These findings suggest that the carrying ability of *NLS* is closely related to the size of cargo proteins. Noted that DENV2-NS5, whose C-terminal 18 residues (Cter_18_) have the same basic aa composition as TMUV *NLS_41–45_*, is fully localized to the nucleus (43). This observation indicates that different cargo proteins may also influence the cellular localization. Indeed, we further confirmed that 3 × GFP could be transported into the nucleus with the assistance of *NLS_41–45_* (typical Class 2 *NLS*), but the similar-sized RdRp domain of TMUV could not. Similarly, both substitutions of functional *α/β NLS* and Cter_18_ in DENV2 did not change the localization of TMUV-NS5. Based on these results, we conclude that, although *NLS* is the region that recognizes and interacted with KPNAs, the subcellular localization of cargo proteins is determined by multiple factors in the nuclear transport system (17, 63), which includes both the size and type of cargo proteins referred to in this study. These also partially explain why JEV-NS5 is not seen in the nucleus, and the modified DENV4-NS5 (in which the *α/β NLS* was replaced with the functional one from DENV2) still exhibits substantially less nucleus-localized than DENV2-NS5 (41, 45).

Mutations in the *α/β NLS* region of DENV2-NS5 impair viral production and block the interaction between NS5 and nuclear transport proteins, attenuating its infectious ability to host cells (60, 64). This indicates that the functional *NLS* is crucial for maintaining *Flavivirus* infectivity. Consequently, *NLS_37–45_* identified currently may be used as a potential target for TMUV treatment, and monitoring variations of basic aa within this region is essential for tracking the changes of TMUV virulence. Thus, our subsequent work will attach more importance to the impact of *NLS_37–45_* on the virulence of TMUV and its antagonism on IFN-I signaling, for example, arginine 888 at Cter_18_ (Class 2 *NLS*) of DENV2-NS5 as the crucial point for RNA replication (43). Taken together, in the current study, we confirmed that TMUV-NS5 interacts with KPNAs via a key region of 37–45 aa in its N-terminus, thereby blocking the nuclear translocation of STATs and functioning as the major inhibitor of IFN-I signaling. We also revealed that the nuclear trafficking capacity of *NLS* is governed by various factors, encompassing but not restricted to the type and molecular size of cargo proteins, thus ultimately dictating the diverse subcellular localization of *Flavivirus* NS5. Furthermore, the effects of *NLS_37–45_* area on TMUV virulence and which point is crucial require further exploration.

## MATERIALS AND METHODS

### Viruses, cell lines, and antibodies

TMUV strain TC2B (GenBank Accession No. MH764605) was previously isolated from the livers of 16-day-old layer ducks from Shandong province in 2011 and preserved in our lab (65). BHK21 (ATCC: CCL-10), HeLa (ATCC: CCL-2), HEK293 (ATCC: CRL-1573), and Vero (ATCC: CCL-81) cell lines were maintained in Dulbecco’s modified Eagle medium (Biological Industries) supplemented with 10% fetal bovine serum (Bio-Channel) and 1 × penicillin-streptomycin (100 U/mL penicillin, 100 µg/mL streptomycin) (Solarbio) in the humidified incubator at 37°C with 5% CO_2_.

Antibodies against TMUV-E and NS5 were prepared in our lab as previously described (66, 67). Commercially available antibodies against STAT1, STAT2, HA, Myc, β-actin, α-Tubulin, Histone-H3, and GFP were purchased from Proteintech. Antibodies against phospho-STAT1 (Tyr701), phospho-STAT2 (Tyr690), and phospho-JAK1 (Tyr1034/1035) were from Cell Signaling Technology. Rabbit polyclonal antibodies against KPNA1, KPNA2, KPNA3, KPNA4, KPNA5, KPNA6, KPNB1, JAK1, IFNAR1, and IRF9 were purchased from Abclonal Technology. CoraLite488-conjugated Goat Anti-Mouse IgG (H + L), CoraLite594-conjugated Goat Anti-Rabbit IgG (H + L), and horseradish peroxidase-conjugated Goat Anti-Mouse/Rabbit IgG (H + L) were also from Proteintech. Recombinant Human IFN-alpha 2 Protein was from NOVUS BIOLOGICALS, and Recombinant Human IFN-β1b was from Beyotime.

### Plasmid constructs

Genes encoding NS proteins and NS5 truncation mutants were amplified from the genomes of TMUV (GenBank Accession No. MH764605) and cloned into the pCAGGS expression vector with an HA-tag in the N-terminus using standard molecular biology techniques. Similarly, plasmids of Myc-STAT1 (GenBank Accession No. BC002704), Myc-KPNA1 (GenBank Accession No. DQ896822), *SV40_NLS_*-2 × GFP (68), 2 × GFP, *NLS_37–45_*−2 × GFP, 3 × GFP, *NLS_37–45_*−3 × GFP, 4 × GFP and *NLS_37–45_*−4 × GFP were constructed and cloned into plasmids pCMV-Myc and pEGFP-C1, respectively. ZIKV-NS5 was chemically synthesized based on GenBank sequence KJ776791. ISRE luciferase (pISRE-TA-luc) and Renilla luciferase (pRL-SV40) reporters were purchased from Beyotime. All constructs were sequenced for confirmation.

### Luciferase reporter assay

HEK293 cells were seeded in 24-well plates and incubated at 37°C with 5% CO_2_ until they reached 70%–90% confluence. Then, plasmids encoding NS1, NS2A, NS2B, NS3, NS4A, NS4B, NS5, or the vector backbone (pCAGGS-HA) were individually co-transfected into cells with 0.1 µg of pISRE-TA-luc and 0.01 µg of pRL-SV40 plasmids using Lipofectamine 2000 (Invitrogen). Twenty-four hours post-transfection, these cells were treated with IFN-α (1,000 U/mL) or -β (1,000 U/mL), and luciferase activities were measured using the Dual-Luciferase Reporter Gene Assay Kit (Beyotime).

### RNA isolation and quantitative real-time PCR

Total RNA was extracted using TRIzol reagent (CWBIO) according to the manufacturer’s instructions, and 1 µg of total RNA was reverse-transcribed into cDNA with FastKing RT Kit (TIANGEN). The relative expression of genes was then measured with qRT-PCR using TB Green Premix Ex Taq (Tli RNaseH Plus) (Takara) on a Lightcycler96 thermocycler (Roche). Transcript levels were analyzed by the 2^−ΔΔCt^ threshold cycle method and are shown as fold induction relative to the levels of the mock-treated control. The housekeeping gene encoding GAPDH was used as the endogenous control to normalize expression levels of the target genes.

### Coimmunoprecipitation and WB

After proper treatments for HEK293 or HeLa cells, co-IP was performed using the Pierce Classic IP Kit (Thermo Scientific) according to the manufacturer’s instructions. The samples were then separated by 12% SDS-PAGE and electroblotted onto PVDF membranes using the wet transfer process. Subsequently, the membranes were blocked in 10% skim milk, incubated with primary and secondary antibodies, and visualized using NcmECL Ultra (NCM Biotech).

### Indirect IFA

Cells were seeded on coverslips, placed in 24-well plates, and incubated at 37°C until reaching 70%–90% conﬂuence. Next, cells were co-transfected with the indicated plasmids for 24 h, fixed in 4% paraformaldehyde for 15 min, and permeabilized with 0.1% Triton X-100 for 10 min at room temperature. After three washes with phosphate-buffered saline (PBS), cells were blocked with 5% bovine serum albumin in PBS for 1 h and incubated with the primary antibody overnight at 4°C. Followed by three washes with PBS, cells were incubated with the appropriate secondary antibody for 1 h at 37°C. Images were observed and photographed by fluorescence microscopy (Nikon) or confocal microscopy (Leica).

### Statistical analysis

All data were analyzed using SPSS Statistics (version 24). Comparisons between two groups were performed using an unpaired two-tailed Student’s t test, while multiple comparisons were performed using one-way analysis of variance with Dunnett’s test. *P < 0.05, **P < 0.01, ***P < 0.001 and no significance (n.s).

## Data Availability

All relevant data are available within the paper.
